# Commensal domestication pathways amongst plants: exploring segetal and ruderal crop origins

**DOI:** 10.1098/rstb.2024.0190

**Published:** 2025-05-15

**Authors:** Dorian Q. Fuller, Tim Denham, Meriel McClatchie, Xiaodi Wu

**Affiliations:** ^1^ Institute of Archaeology, University College London, London, UK; ^2^ School of Cultural Heritage, Northwest University, Xi'an, Shaanxi, People’s Republic of China; ^3^ Archaeology and Anthropology, Australian National University, Canberra, Australian Capital Territory, Australia; ^4^ School of Archaeology, University College Dublin, Dublin, Republic of Ireland

**Keywords:** phenotypic change, evolution rate, archaeobotany, conscious selection, unconscious selection, weeds

## Abstract

Two commensal pathways to plant domestication—ruderal and segetal—have been proposed. These domestication pathways are detailed here, together with associated archaeobotanical morphometric data for multiple crops within each pathway. The ruderal pathway characterizes how plants adapted to anthropically disturbed habitats, which can be associated with foraging or farming communities, were domesticated by people. Ruderal crops discussed are squash (*Cucurbita pepo*), aji chili (*Capsicum baccatum*) and melon (*Cucumis melo*). The segetal pathway characterizes how weeds in agricultural contexts became crops. Segetal crops discussed are rye (*Secale cereale*) and kodo millet (*Paspalum scrobiculatum*). Metric archaeobotanical datasets are used to infer the domestication episode for crops and to calculate rates of change in domestication traits (Haldanes). Although metric archaeobotanical data limits presentation and discussion to seeds, it enables quantitative comparisons of domestication episodes and haldane rates with those of the grain and fruit tree domestication pathways, respectively. We conclude that early ruderals underwent slower domestication processes, whereas segetals and perhaps some later ruderals, underwent faster processes of domestication that probably involved conscious selection.

This article is part of the theme issue ‘Unravelling domestication: multi-disciplinary perspectives on human and non-human relationships in the past, present and future’.

## 1. Introduction

Plant domestication has most often been characterized and documented archaeologically for seed crops that followed a grain domestication pathway, such as major cereals. For the domestication of cereals, wild plants were brought under human control through cycles of seed propagation and storage leading to selection for a suite of domestication syndrome traits, including non-shattering, larger seeds and loss of dormancy. However, several other pathways of plant domestication have been proposed (as per Fuller *et al.* [1]), for which there is less botanical and archaeological documentation of the domestication process and limited understanding of how plants are entangled with practises in fields and the wider landscape. In the present contribution, we detail and present archaeobotanical data for two distinct commensal plant pathways that have only been previously proposed in abbreviated form: segetal pathway (plants that originally grew as volunteer weeds within cultivation) and the ruderal pathway (plants that benefitted from anthropogenic disturbance in and around human activity areas). We explore differences in the anthropogenic niches in which these commensals grew and the practises that brought them into intentional cultivation through which domestication syndrome traits evolved. We also consider how these pathways vary from each other and from the primary grain domestication pathway that has been more frequently documented in archaeobotanical studies (e.g. [1–4]). We establish working hypotheses about how the rates of evolution of domestication traits and selection pressures differed among the segetal, ruderal, and grain pathways to domestication.

## 2. Domestication traits and pathways

Archaeological studies of plant domestication have focussed on tracking morphotypic changes in archaeobotanical assemblages, such as increasing proportions of non-shattering spikelet bases, larger grain sizes and thinner seed coats in cereals and pulses (e.g. [2,4–10]). These types of archaeobotanical studies provide invaluable data on the domestication episode, rates of change in traits (Haldanes), locations of early domestication and regions of subsequent spread [3,4]. Although these domestication traits are convergent in many sexually propagated grain crops, they comprise a domestication syndrome of phenotypic traits that are shared by many cereals and pulses [5,11,12]—these traits are not necessarily common to other types of domesticated crop plants. For instance, non-shattering, seed size and seed dormancy are not significant domestication traits in vegetatively propagated crops, which are clonally propagated from a variety of plant parts, such as a stem or root fragment, cutting, sucker and so on [13,14]; indeed, in some clonally propagated fruits—such as bananas [15] and breadfruit [16]—extreme seed suppression has resulted in major cultivar groups having vestigial seeds, effectively being ‘seedless’. Consequently, a range of alternative phenotypic traits are required to track the domestication of these plants in the past [13,17].

An awareness of the diversity of the potential domestication traits in different types of crops has increased awareness of the diversity of human–plant domesticatory relationships through which different types of plants were domesticated. There has long been a recognition that morphotypic domestication as visible in the archaeobotanical record may in some contexts be a somewhat arbitrary marker of the reliance of people on a given plant for subsistence or of the intensity of its management and cultivation [18]. Some types of plant may exhibit greater plastic adaptation to growth environments than others, or the traits may not be preserved in an identifiable phenotypic form [13,19,20]. Some communities may rely heavily on the cultivation of morphologically wild plants, namely ‘cultiwilds’ [21] and semi-domesticated plants [22] for extended periods [23,24]. In seeking to understand the diversity of human–plant domesticatory relationships in different parts of the world, we need to acknowledge the diversity of domestication pathways, considering each of them in their own terms [18] and as stable end points [25]. In doing so, our interpretative emphasis expands from a focus on plant domestication traits *per se* (namely, archaeobotanical data) to contextualizing these data within pathways that contextualize domestication in terms of parallels in human practises of habitat shaping (namely, human–plant domesticatory relationships).

Seven discrete pathways of plant domestication have been proposed (table 1). Just as with Melinda Zeder’s pathways to animal domestication [26,27], each pathway seeks to differentiate the practises of exploitation, management, selection, reproduction and cultivation through which types of plants were domesticated. Although each pathway is different, they are not mutually exclusive or rigid categories, rather they are heuristic devices to characterize key aspects of human–plant domesticatory relationships through time. In order to ensure interpretative equivalence, a common conceptual framework has been developed to characterize the qualitatively different domestication pathways [1,14].

**Table 1.** Pathways to plant domestication (amended from [1]). (These pathways are not exclusive, rather they represent commonalities in the ways that plants were domesticated by people in different parts of the world in the past.)

pathway	description	example crops
ecosystem engineering	focus on long-lived trees, palms and pandanus within managed ecosystems	brazil nut (*Bertholletia excelsa*) oil palm (*Elaeis guineensis*) pandans (*P. conoideus/julianettii*) sago palm (*Metroxylon sagu*)
ruderal	plants that adapt and spread within disturbed, anthropic environments, such as camp-following commensals; need not initially be associated with cultivation in plots/fields	bottle gourd (*Lagenaria siceraria*) cane grasses (*Saccharum* spp.) chili peppers (*Capsicum* spp.) melon (*Cucumis melo*) squash (*Cucurbita* spp.)
tuber	plants that yield starch-rich underground storage organs that are reproductively viable and can be used for vegetative propagation of the crop	manioc/cassava (*Manihot esculenta*) potato (*Solanum tuberosum*) sweet potato (*Ipomoea batatas*) taro (*Colocasia esculenta*) yams (*Dioscorea* spp.)
grain	annual crops grown for/from seeds (especially cereals and pulses)	barley (*Hordeum vulgare*) lentil (*Cicer arietinum*) maize (*Zea mays*) pea (*Pisum sativum*) rice (*Oryza sativa*) soybean (*Glycine max*) wheat (*Triticum* spp.)
segetal	former weedy species that grew in agricultural contexts which were added to the crop repertoire (can also be important for fodder)	Indian kodo millet (*Paspalum scrobiculatum*) kudzu (*Pueraria lobata*) oats (*Avena sativa*) rye (*Secale cereale*) West African fonios (*Digitaria exilis*)
fibre	directed selection of species that were already crops or weeds, but subsequently grown for fibre and not food	cotton (*Gossypium* spp.) flax (*Linum usitatissimum*) hemp (*Cannabis sativa*) jute (*Chorchorus* spp.)
fruit tree	woody, longer-lived perennial species grown for fruit that are added to existing grain or tuber-based economies; initially planted from seed (often for an extended period) and then often propagated using cuttings/grafting/vegetatively	citrus (*Citrus* spp.) date palm (*Phoenix dactylifera*) fig (*Ficus carica*) grape (*Vitis* spp.) olive (*Olea europaea*) peach (*Amygdalus persicus*)

Initially, the divergence between grain and tuber pathways was detailed, given the clear discrepancies between modes of plant reproduction, cultivation practises, utilized plant parts and potential domestication syndrome traits [4,13,28]. Plants with underground storage organs that are reproductively viable—collectively connoted by the ‘tuber pathway’ here—were probably initially exploited and managed in ad hoc ways in gaps and anthropic patches, such as within forests, then translocated and grown in polycultural plots [24]. Comparably, grains initially underwent management and resource intensification within the landscape, such as riparian corridors and semi-arid landscapes, potentially with broadcasting of seed within the environment to expand wild resource availability, before sowing in especially created, tilled plots [29,30]. In tuber and grain pathways, there is an increasing separation of growth environment from ecosystem through time, with domestication following cultivation in plots.

The fruit tree pathway has also previously been characterized, entailing a switch from sexual to asexual reproduction (grafting, transplanting), selection for reduced interannual variability, reduced outcrossing, lengthened seeds with more fruit flesh and higher sugar content [14,31–33]. Fruit trees were domesticated from woody perennials and probably augmented pre-existing cultivation practises involving grains or tubers. Similarly, fibre crop pathways tend to be directed by adding non-food crops to agricultural systems or selecting high-fibre production from crops initially grown for multiple functions, as seems to be the case with flax and hemp [34]. Ecosystem engineering entails domesticatory relationships occurring within and throughout the ecosystem or environment, and has been best-characterized for tropical rainforest environments [35,36]. This may involve minimal evolution of changes in individual species that we recognize as domestication traits.

**Figure 1.**
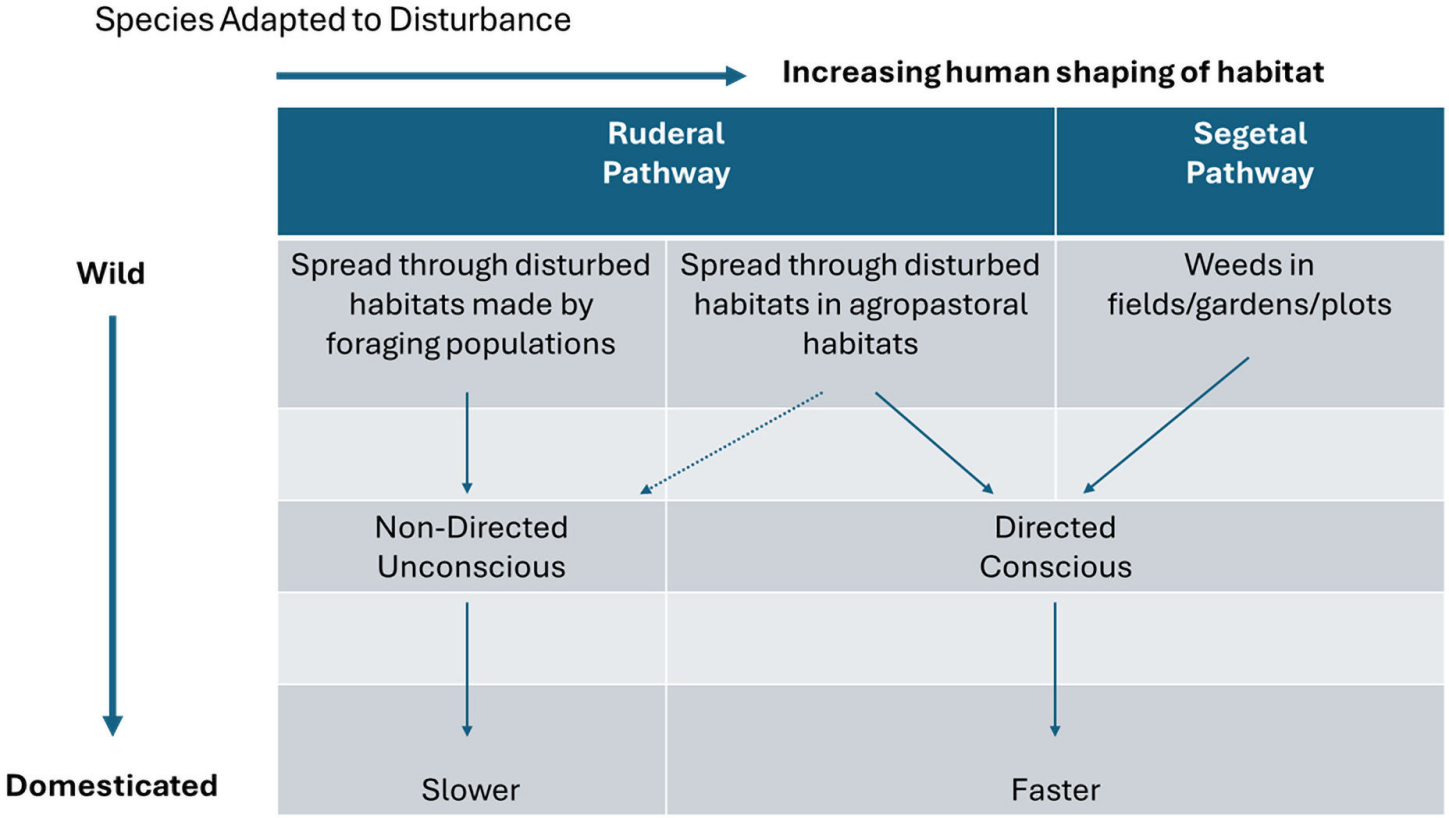
Conceptualizing segetal and ruderal pathways along a spectrum from lesser (forager) to greater (farmer) habitat shaping, following alternative domestication trajectories in terms of evolutionary rate and degree of consciously directed selection.

The two pathways of interest in the present study focus on different kinds of commensals (figure 1). All such species have some pre-existing adaptations to habitat disturbance, but this can vary from clearing, trampling and middening through human settlement activities to the more systematic soil disturbance of arable fields. Ruderals are plants that initially benefitted from disturbed niches within the ecosystem created by people, such as locally cleared areas and rubbish heaps around camp sites; discarded seeds or asexually reproductive parts were able to grow and the plants spread together with people as they moved through the landscape. Segetals were already part of the agricultural landscape when they began to be domesticated. Segetals are arable weeds that thrive in fields and compete with crops and thus were already evolving in arable systems before they became a focus of such systems. Neither the ruderal or segetal pathway has been characterized in detail previously, nor considered with respect to archaeobotanical evidence for extent or rate morphological change in the plants involved.

Domestication pathways can also be conceptualized in terms of the degree of intended, deliberate, purposeful or conscious behaviour to bring about desired phenotypic traits. The initial periods of ecosystem engineering, ruderal, tuber and grain pathways would largely have been the exploitation and *in situ* management of desired plants, yet with unforeseen long-term consequences, such as the increase of plant resources in the landscape. Through time, people increasingly disturbed environments—with clearing, burning and transplanting propagules/broadcasting seed—to enable the growth of favoured phenotypes for desired size, taste, colour, palatability, lesser toxicity, abundance and so on. However, the long-term and cumulative consequences of these practises could not have been fully anticipated by individual communities, as the processes of phenotypic transformation leading to domestication occurred over millennia. As plants were increasingly planted in prepared plots, selection became more conscious. By contrast, the domestication of segetals, fibre crops and fruit trees involved an intentional process of diversifying cultivation by communities who were already farmers and who brought these plants into their crop repertoire for specific reasons: this raises the possibility of conscious selection for certain traits.

At the global scale, recognition of the qualitatively different, yet conceptually equivalent, domestication pathways highlights the diversity of plant domestication practises in different regions. These domesticatory relationships occurred over extended time periods, not just the time frame after which domestication traits had been ‘fixed’. Rather a focus on pathways draws attention to the often long time periods of human–plant domesticatory relationship preceding clear and cumulative, or ‘fixed’, morphotypic domestication. From this perspective, domestication is conceived as a temporal process, an episode, rather than a point in time. The domestication pathways for ecosystem engineering of tropical rainforests [36], as well as many ruderals, e.g. *Cucurbita* spp., *Lagenaria siceraria* [4,37], some grains [4] and some tubers [24], stretch back into the early Holocene, if not earlier. For fibre crops, there are few that have well-documented domestication episodes. Some, like cotton, were cultivated initially for their fibres and this may have begun in the earlier Holocene in both the Americas and South Asia [38,39]; others, such as flax and hemp may have differentiated into fibre-selected varieties after earlier multi-use domestication that included cultivation for human consumption [34]. By contrast, domestication episodes for segetals, as we illustrate below, and fruit trees mostly occur in the mid and late Holocene. However, formal domestication in all pathways occurred much later than the beginning of the domesticatory relationship (initial management or cultivation).

## 3. Material and methods

In the present paper, we explore the segetal and ruderal pathways through a selection of species. For each pathway, we present a qualitative interpretation of the human–plant domesticatory relationships through time together with a quantitative assessment of the durations and rates of change associated with the domestication episodes for specific species. The available quantitative archaeobotanical data are highly variable in completeness for each species, yet sufficient to allow us to identify the period of the domestication episode and to quantify the amount and rate of change, particularly in seed size. For the segetal pathway, we focus on rye (*Secale cereale*) from Europe and kodo millet (*Paspalum scrobiculatum*) from India. For the ruderal pathways we consider Mesoamerican squash (*Cucurbita pepo),* South American aji chili (*Capsicum baccatum)* and East Asian melon (*Cucumis melo)*. The findings are discussed in comparison to measured rates of domestication for several cereal crops within the grain pathway.

Datasets for charting grain size evolution were compiled in terms of mean, standard deviation, maximum and minimum metrics. Previous work in cereals found that grain breadth and thickness changed most markedly with domestication and generally at similar rates [3,4,40]. Therefore, in this study, we focused on grain breadth for each species. In the case of kodo millet (*P. scrobiculatum*), we also explored combining length and width into an area size estimate. As in these previous works, we plotted archaeological metrics on a timeline based on estimated median age, and whenever possible this was derived from the summed probability of all radiocarbon dates available from a site phase (see discussion of dating in [4,40]). Where standard deviation was not reported in the original source this was estimated based on sample size and range, assuming a normal distribution, using the denominator in table 27 of Pearson & Hartley [41]. Domestication episodes were identified in data plots visually as the first period of directional increase; in some cases, shorter and longer episodes were estimated for the same species.

An evolutionary rate, or rate of phenotypic change was calculated using the *Haldane unit* [42], as this has proved informative in other domestication studies [3,4,33]. The Haldane value represents the rate of measured change in a trait’s quantitative values per generation (assumed to be 1 year for annual plants considered here, but longer for tree crops, see [33]). The Haldane unit itself is a shift of the mean by a standard deviation unit; but the resulting calculations are fractions of such a shift because nothing is expected to evolve by an entire standard deviation in a single generation. This is part of the class of rate estimates that Hunt has termed ‘traditional rates’ [43]. A comparison of this with other approaches to estimating rates of phenotypic evolution suggests that the Haldane rate is especially appropriate for highly directional evolution [43], which is what is expected under domestication. Although the palaeontological record may suggest that directional selection over geological time periods is rare [44], it is an appropriate assumption for domestication as plants shift across the adaptive landscape from the potential stabilizing selection on wild progenitors in nature to a new peak in the adaptive landscape represented by cultivation (on adaptive landscape and directional selection, e.g. [44]). Use of Haldane rates has the advantage that it allows direct comparison to previously published studies of domestication that have used this metric.

Further details of the datasets and their sources are described in the electronic supplementary material. Most data are gathered from published literature but similar time series data have never been compiled for *P. scrobiculatum* or *S. cereale* previously. The *P. scrobiculatum* is based on measurements of archaeological specimens carried out in London and reported here, to our knowledge, for the first time. While previous rate estimates have been published for *Cucumis melo* and *Curcurbita pepo* [4], these are augmented here by additional data points providing updated estimates. For *Ca. baccatum,* we offer the first evolutionary rate estimates based on combining two previous studies of size time series.

## 4. Results and discussion 1: the ruderal pathway

Ruderal pathways include some of the earliest plant domesticates selected from amongst ‘camp-following weeds’, those plants that grow up from discards near human occupation sites, such as bottle gourds (*L. siceraria*), squash (*Cucurbita pepo*) and watermelon (*Cucumis melo*), that preceded early agriculture, as well as later, highly valued crops, such as chili (*Capsicum* spp.), coca (*Erythroxylum* spp.) and cane grasses (*Saccharum* spp.). Although most commonly associated with inadvertent domestication in forager contexts, they also seem to have been domesticated more deliberately in farmer contexts, most likely including both vegetatively and seed-reproduced plants. As yet, we do not have quantifiable datasets on any vegetatively propagated ruderal species to infer rates of domestication, rather domestication histories are based on qualitative multidisciplinary consilience, such as for cane grasses (*Saccharum* spp. [45]). Here we focus on squash (*Cucurbita pepo*), aji chili (*Ca. baccatum*) and Chinese melon (*Cucumis melo*).

### (a) Squash (*Cucurbita pepo*)

The Cucurbit family has 10 important domesticated species, some of which were probably domesticated more than once (e.g. *L. siceraria, Cucurbita pepo, Cucumis melo*) [46]. All of these taxa have edible fruits, although some must be consumed immature, and they have seeds that have oily inner kernels that may be edible directly or processed for oil. It is likely that use of wild plants, including by hunter-gatherers could have concentrated discarded seeds of these species around areas of human visitation, from which new wild stands in anthropogenic habitats sprung up and were exploited.

Some cucurbits have mature fruits that are used for purposes other than food, such as bottle gourd as a container. Bottle gourd is inferred to be amongst the earliest plant domesticates in tropical America and parts of East Asia [47,48]. Container use of the bottle gourd is indicated in selection for thickened rinds. While immature and thin-rind fruits might have been eaten, thicker-rind fruits selected as containers would have had seeds scraped from their interior thus selecting increasingly thicker-rind genotypes in spontaneous populations around encampments and human activity areas.

*Cucurbita pepo* gourds when mature may be dried and used as small containers, or as suggested in North America for fishing net floats [49,50]. When cultivation for food took place, however, we expected selection for larger fruits with more flesh, which is linked to an increase in seed size. In North America, larger seed sizes outside the natural range of wild *Cucurbita pepo* are present at the Phillips Spring site in southwestern Missouri dated to 5000 BP [37]. This is the earliest food plant with evident morphological domestication amongst those found in the eastern woodlands of North America (or the Mississippi basin), with other grain crop taxa showing domesticated morphologies about 1000 years later.

**Figure 2.**
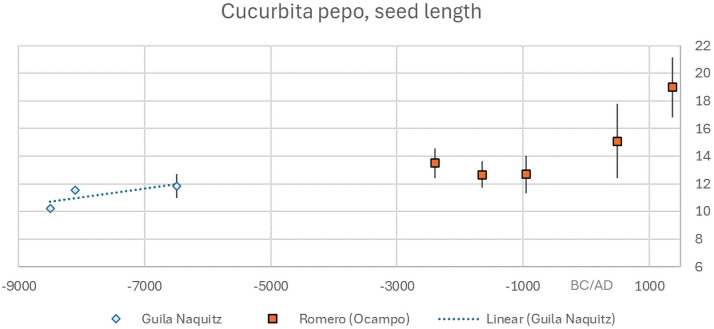
Archaeobotanical seed size data plotted against median age for *Cucurbita pepo* from Mexico (data: electronic supplementary material, table S1).

**Figure 3.**
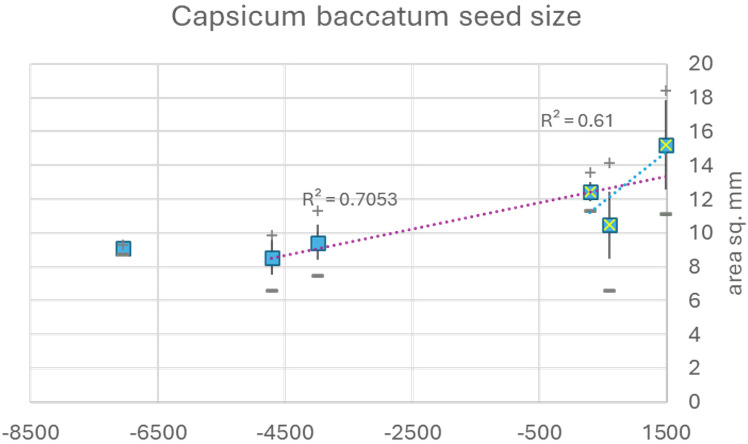
Archaeobotanical assemblage seed size (diameter/length) data for *Capsicum baccatum*, indicating two alternative domestication trends (early/slow versus late/fast).

*Cucurbita pepo* has a separate and earlier domestication in Mesoamerica. The separate domestication is well-evidenced in genomic data [51]. Smith [37] has pointed to evidence for domestication in the appearance of thicker penduncles in desiccated assemblages from Mexican caves, especially Guila Naquitz, as well as increasing seed size. These data have been replotted on a timeline together with data from Ocampo Caves (figures 2 and 3), which shows a marked seed lengthening averaging *ca* +15% between 10 000 and 8000 BP. There is a more modest increase over subsequent millennia, until the most recent 1500 years when size increases again. The sample size available during the proposed domestication episode is too small to be robust, yet if substantiated would provide a Haldane rate calculation of approximately 0.9 × 10^−3^, which is comparable to that in primary cereals. We interpret this to suggest an unconscious process of evolution within ruderal populations, from which people eventually began to intentionally plant fruits that had already evolved larger sizes, perhaps through a process of competition amongst seedlings (as per [52]). This domestication process precedes that for maize (*Zea mays*), for which the first phytolith indicators of potential domestication traits date to *ca* 8700 BP [53] and for which the earliest cobs large enough to support agricultural-based sedentism are inferred to occur only around 3700 BP [54]. Thus while *Curcurbita* domestication may have occurred alongside the cultivation of other plants in both North America and Mexico, its domestication appears earlier than that for other crops in both regions.

### (b) Chili (*Capsicum baccatum*)

Although the best documented of the five chili pepper domestications (*Capsicum* spp.), there is only limited quantitative archaeobotanical data for *Ca. baccatum* (figure 3). Microfossil starch analyses have suggested an early domestication and dispersal of chili peppers within the Americas [55], but such early finds are not complemented by robust macrobotanical assemblages of seeds. Seeds of *Ca. baccatum* older than about 6000 BP have mean diameters of 9.5 mm, after which there is a long gap in data until the third/fourth century AD (approx. 1700 BP) when seed diameters are typically over 10.5 mm and assemblage averages are approximately 12.5 mm. It is unclear when the size of chili peppers increases within this more than 4000 year period. If domestication began just after our *ca* 6000 BP datapoint, then we can envisage a very protracted and slow domestication process—longer and slower than a typical primary cereal—0.5×10^–3^ Haldanes. By contrast, another estimate can be derived from later datapoints (from 1700 BP to pre-Colombian 1491 AD) that produces a 1.1×10^–3^ Haldane, which is much more typical of other domestications. In the absence of robust archaeobotanical data, we propose that *Capsicum* spp. are ruderal domesticates, namely, they were originally wild harvested in disturbed contexts around human sites and then domesticated and systematically cultivated probably after the establishment of agriculture based on other species.

### (c) Melon (*Cucumis melo*)

**Figure 4.**
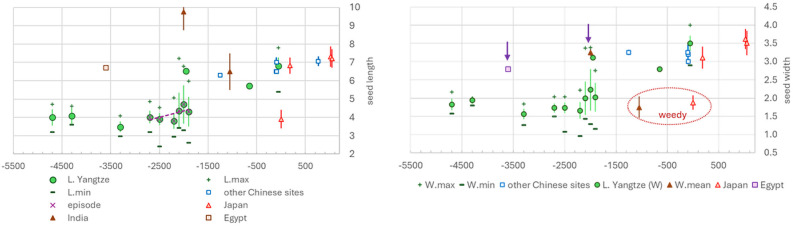
Archaeobotanical seed size data from *Cucumis melo*. In the left graph, seed length is plotted against median age estimate, with indication of inferred domestication episode in the Lower Yangtze region of China. In the right graph, seed width is similarly plotted with arrows indicating early large, and hence domesticated, examples from early Egypt and the Indus region that support three independent regional domestication processes; also labelled are late occurrences of inferred wild-type or weedy melons from India and Japan.

The wild progenitors of melons derive from the complex of *Cucumis collosus*, *Cucumis trigonus* and *Cucumis melo* subsp. *agrestis,* which are generally taken as synonyms [56–58]. *Cucumis colossus* has been described as truly wild [57] while *agrestis* wild melons prefer arable habitat margins and may include feral melons [59–61]. *Cucumis melo agrestis* is also found in parts of East Asia, including central and eastern China, where it is common in disturbed settings, including agricultural fields [62]. A study in South Korea found that *Cucumis melo agrestis* could often be found on the margins of rice fields, in the fields of soybean and sweet potato, as well as in manure piles and roadsides [63]. Many authors argue that there were two or three parallel domestications of melon [58,61] in India [57], in Egypt from African wild melons [61,64] and in China, probably represented by the Lower Yangtze archaeological evidence [65,66] (figure 4).

The time series of metrical data from the Lower Yangtze shows a domestication episode starting shortly before 4000 BP. Prior to this, with samples going back *ca* 7000 BP, seeds averaged around 4 mm or less in length and 1.5–2 mm in width (figure 4), but there is marked increase sometime after 4300 BP. The domestication episode itself is 500–600 years long. This gives an estimated rate of change in Haldanes of 2.6×10^–3^, about twice as fast as cereals [3,4], and half as fast as segetal crops like *Secale* and *Paspalum* (see below). Then in later periods after 4000 BP, many seeds reach lengths of 6–7 mm and of over 3 mm width. The idea of independent domestications in Egypt and the Indus region is supported by large, domestic-size seeds by this time or earlier, i.e. 5600 BP at Maadi in Egypt [67] and by 4000 BP in the Indus [68].

The cultural setting of melon domestication in China was the Liangzhu culture, known for its specialized craft production in jade, ceramics and textiles, as well as for wet rice production [69–71]. In this context, melons would not have grown as weeds in rice fields but might have been found in marginal anthropogenic settings that were drier such as settlement margins. Harvesting and inadvertent sowing from such settings may have facilitated the evolution of variability in fruit size and variability along the bitter-sweet spectrum until such time as intentional garden planting of larger, sweeter-fleshed varieties took place and kickstarted a rapid domestication process.

## 5. Results and discussion 2: the segetal pathway

Segetal pathways include ‘secondary’ cereals that were domesticated from existing field weeds, such as oats (*Avena sativa, Avena chinensis, Avena abyssinica,* in Europe, China and Ethiopia, respectively), West African fonio (*Digitaria exilis*), Indian kodo millet (*P. scrobiculatum*), Asian fathen (*Chenopodium album)* and perhaps buckwheat (*Fagopyrum esculentum*). While these share the same domestication syndrome traits as ‘primary’ cereals (reduced shattering, reduced dormancy, denser panicles and compact growth habit, larger grains), segetal domesticates originally evolved as highly successful competitors within weed assemblages that hitchhiked with dispersing agro-ecosystems.

### (a) European rye (*Secale cereale*)

Rye (*S. cereale*) is a classic example of secondary domestication from a weed plant. Vavilov [72] originally defined secondary domestication on the basis of his observations and historical inferences of rye and oat (*A. sativa sensu lato*). He argued that both, which can be found as weeds well beyond their inferred West Asian centre of origin, spread as weeds in fields of wheat and barley. In places where these weeds outcompeted the cereal crops, they eventually came to be cultivated as crops in their own right and subsequently evolved a full set of domestication traits. Vavilov inferred this process from observations in Afghanistan and in Soviet Central Asia where with increasing elevation there were shifts from winter wheat with *Secale* as a weed, to *Secale* as a highly competitive weed, to only rye as a domesticated crop at elevations above the altitudinal range of wheat. At these higher elevations, rye was non-shattering (i.e. domesticated). In addition to being adapted to higher elevation, rye does well under cooler weather at higher latitudes. The derivation of rye from an arable weed-adapted form (*Secale vavilovii* in conventional taxonomy) is strongly supported by recent genomic studies, which also demonstrates continued introgression between crops, weeds and the wild populations in West Asia [73,74].

Currently, there is no consensus on the location where domesticated rye evolved, but it is generally accepted that rye spread out of Anatolia as an arable weed into Europe during the Neolithic more than 6000 years ago [64,73,75,76]. Weedy rye also presumably spread north into the Caucasus and eastwards towards Iran and Afghanistan, where it was observed to be so diverse by Vavilov in the 1920s [72].

Archaeobotanical evidence for rye is biased towards Europe where more work has been done. Finds in Neolithic central Europe are occasional and consist of very low numbers of grains, raising the possibility that some are intrusive grains from later periods. For example, a single grain found at the Neolithic site of Wach ow Niebede in Northern Germany has dimensions, such as a width of 2.7 mm, which are large enough to suggest domesticated rye [77], yet the photograph of the grain indicates blistering which could mean that it has puffed and expanded when charred; it had previously been regarded as a Neolithic weed [77]. At Neolithic Cuneşti, Romania, which dates to 6600−5900 BP, 12 rye grains were recovered in the 1950s and often accepted as the earliest rye of the European Neolithic, yet, recent direct Accelerator Mass Spectrometry (AMS) radiocarbon dating of two seeds showed they were intrusive and derived from the twelfth and fourteenth centuries AD [78]. A similar pattern has been found with millet (*Panicum miliaceum*), in which occasional finds from apparent Neolithic contexts have come to be regarded as intrusive following direct AMS radiocarbon dating programmes that confirm the earliest grains in Europe and the Caucasus region occur from *ca* 3600 BP onwards [79,80].

Systematic reviews of *Secale* finds, including Behre’s [75] review of western Europe, Gyulai’s [76] review of the Caparthian region (especially Hungary), and Grikpedis and Motuzeite Matuzevičiūte’s [81] study of northeastern Europe conclude that there is scant evidence for domesticated *Secale* before the Late Iron Age or Roman period. In the Baltic region of Europe, the earliest well-dated rye (with a domestic-type grain breadth of approx. 1.9 mm) comes from Gabrieliškes hillfort, Lithuania, dated to the first to third century AD, approximately 1800 BP. In Hungary, increasing occurrences of *Secale* grains are reported from the centuries after 2400 BP (Late Iron Age), but typically these are noted to be thin (wild-like) even when they occur in their hundreds [76], as opposed to much larger quantities of larger grains in the early centuries after 2000 BP. Earlier Iron Age finds up to *ca* 2200 BP were often only a few grains, whereas from the 1800 BP larger finds in terms of quantity began to appear in central Europe, e.g. Germany and Austria [75]. While Behre [75] pointed to a potential centre around the Black Sea from which cultivated rye spread, the available metrics compiled herein show an increase in mean grain breadth appearing in the last three centuries BC (after 2300 BP) in Germany and Denmark, France by 2400 BP; and in Portugal at the Roman era site (third to first century BC) of Crastoeiro [82]. Thus on available evidence it is plausible that rye domestication started in western Europe *ca* 2500−2000 BP.

**Figure 5.**
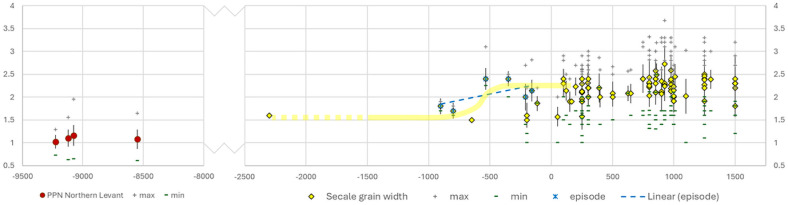
Archaeobotanical grain size data plotted against median age for *Secale.* In the left graph, an outgroup baseline from Pre-Pottery Neolithic (PPN) southwest Asia (Syria); in the right graph, measurements from European archaeological assemblages with an inferred domestication episode indicated in the first millennium BC.

Figure 5 plots archaeobotanical measurements from across Europe, alongside an outgroup baseline of earlier wild-type rye from southwest Asia. These early finds of *Secale strictum,* which may include some *S. vavilovii* grains [83,84], are regarded to be early cultivars, and some larger grains and non-shattering rachises appeared before 9000 BP at Abu Hureyra to support the case for a domestication process [83]. However, rye never became a widespread crop of the Neolithic Near East and very probably was extirpated from cultivation during the Pre-Pottery Neolithic, even though it seems to have spread to Can Hassan III in Central Anatolia by *ca* 9500 BP [85], but direct dating to confirm this is not available. Early grain metrics from Syria indicate an approximately 13% increase in mean breadth over about 700 years. *Secale strictum* are expected to be somewhat smaller (thinner) than weedy annuals (especially *S. vavilovii* subsp. *vavlilovi*), the immediate ancestral complex to domesticated *S. cereale* [73,86].

All finds in Europe from the Bronze Age onwards are assumed to represent either the weedy (*S. vavilovii*) or intentionally cultivated forms of *S. cereale.* In terms of available measurements, we can identify an episode of grain size increase in *Secale* over the first millennium BC; thinner grains occur at sites older than *ca* 3000 BP and wider grains occur from 2500 BP. This suggests a domestication episode of 500–1000 years, exhibiting a mean grain breadth increase of +29%. In terms of Haldane rates, a shorter 550 year episode produced a Haldane rate of 6.5 × 10^–3^, whereas a longer 750 year episode gave a Haldane rate of 2.2× 10^–3^. The lower rate is about double the estimates for wheat and barley grain size increase [3,40], whereas the shorter episode is six times faster.

### (b) Kodo millet (*Paspalum scrobiculatum*)

Kodo millet is one of four widespread indigenous millet crops in India (others being *Brachiaria ramosa, Echinochloa frumentacea* and *Panicum sumatrense)*. Although its domestication in India has long been recognized [87,88], this has not been studied archaeobotanically in terms of either when and where it emerged or in terms of documenting morphological changes. Observations have suggested reduced panicle shattering and fatter/thicker grains characterized the crop compared to wild relatives [87], although this has not been systematically documented. Recent efforts to improve this crop through modern breeding have highlighted that yield related traits have substantial heritability, estimated as approximately 66% for grain weight [89]. Selection for alleles that increased grain size probably took place during the domestication process, as has been documented for numerous crops [1,52].

Of all the indigenous Indian millets, *P. scrobiculatum* presents the strongest case for a secondary domestication process following a segetal pathway. Some have speculated that the minor Indian millets were all secondary crops (our segetal pathway) that originally occurred as weeds in either wet or dry rice fields [90]. However, archaeobotanical evidence makes it clear that *B. ramosa* was the dominant staple cereal in the Neolithic of south India by *ca* 4000 BP [91–93], whereas rice was not introduced to the region until sometime after 1000 BC [94]. In Gujarat, western India, early agriculture around 2500 BC included *B. ramosa* but was dominated by *Panicum sumatrense,* which long predates the introduction of domesticated rice to the region around 3000 years ago [88,95–97]. By contrast, *P. scrobiculatum* occurs rarely and in very small quantities at early farming sites in these regions, perhaps as an occasional weed of other millets [91,98], and on Neolithic/Chalcolithic sites (4000−3000 BP) in the Ganges plains were it is plausibly a weed of early rice fields [99,100]. Where quantitative data are available *Paspalum* occurs in just single grain finds and often in single samples [101].

**Figure 6.**
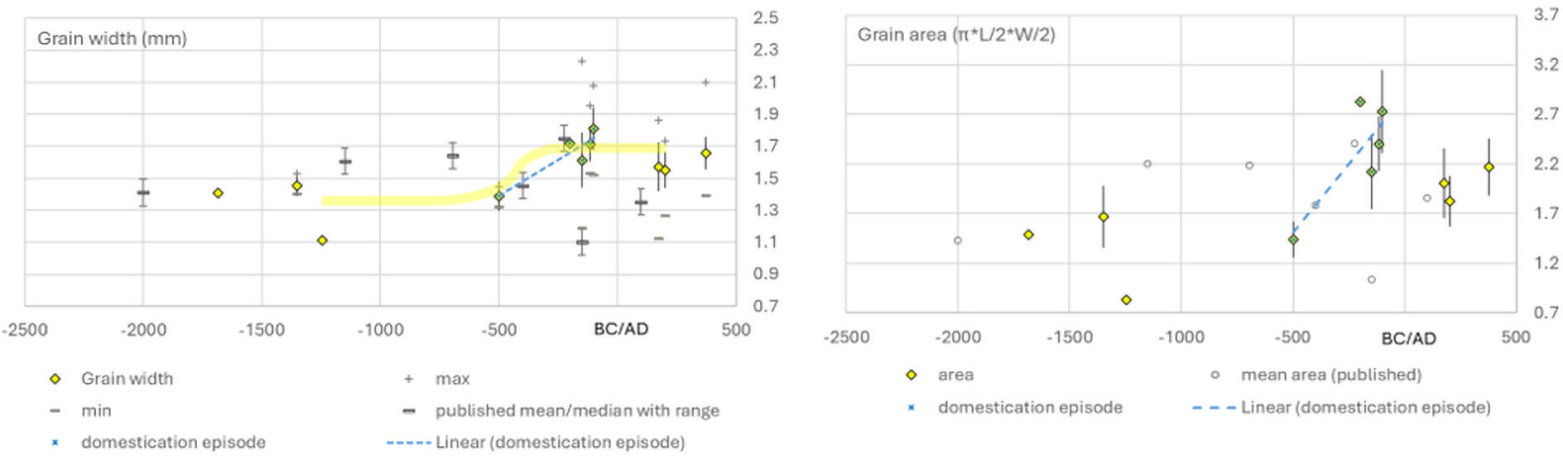
Archaeobotanical grain size data plotted against median age for *Paspalum scrobiculatum* in archaeological assemblages from India and Sri Lanka, indicating inferred domestication episode. The left graph plots grain width, the right graph plots elliptical area calculated from grain length and width.

At Iron Age sites in south India (perhaps in the 2700−2300 BP range) and Early Historic sites (after 2300 BP) in south and north India, kodo millet occurs in larger quantities and numerous samples, e.g. Veerapuram [102], Hulaskhera [103] and the sites with measurements reported in this paper. This period of increased occurrence of *P. scrobiculatum* coincides with the domestication episode, indicated by the marked increase in grain size between 2500 BP and *ca* 2100 BP (figure 6). Although limited in quantity archaeological *Paspalum* grains up to 2500 BP are all less than 1.7 mm wide and mostly below 1.5 mm, while elliptical area estimates of grain size are mostly below 2 mm^2^. After the domestication episode, by the centuries before 2000 BP average grain breadths are above 1.6 mm and with larger grains often being above 2 mm. Precise dating of when this episode began is hampered by a lack of sites dating to this period and difficulties with radiocarbon during the Iron Age calibration plateau (2750−2400 BP). Veerapuram, which occurs within this period and has numerous reported kodo millet grains contains larger and thicker grains that are probably early indicators of selection on grain size [102]. Actual measurements from Veerapuram were not reported and are therefore ripe for re-study.

Based on the available archaeobotanical evidence, we can tentatively estimate that the domestication episode for kodo millet lasted 500 years, perhaps less. Such a rapid change contrasts with early cereal domestication in west Asia which took place over at least 2000 years, if not longer (see [1,4,10]). In the estimate of the rate of phenotypic change in terms of Haldane units, *Paspalum* was domesticated about five or six times faster than cereals such as wheats, barley or rice (table 2). This higher rate of evolution is comparable to rye, especially using the shorter 550 years domestication episode for that crop (see above). Taken together these two segetal cereal domestications support shorter (<1000 years) and faster phenotypic evolutionary rates for this pathway.

**Table 2.** A comparison of evolutionary rate estimates (in Haldanes) for ruderal and segetal taxa (this paper) with those previously estimated for a selection of primary grain crops [4] and tree fruits [33]. Note estimates in [4] were published with an error in the formula, which has been corrected here.

species	path	trait	episode	samples size (for episode)	% change	% per year	Haldanes
*Hordeum vulgare*	grain	non-shattering	9080−6200 BC	7854	96.0%	0.03%	0.6×10^–3^
*Hordeum vulgare*	grain	grain breadth	9500−6950 BC	578	46.1%	0.01%	1.1×10^–3^
*Triticum monococcum*	grain	non-shattering	9725−6660 BC	1441	94.6%	0.03%	1.2×10^–3^
*Triticum monococcum*	grain	grain breadth	9725−6450 BC	1064	44.0%	0.01%	1.2×10^–3^
*Oryza sativa*	grain	grain width	5800−3700 BC	1489	29.8%	0.01%	0.5×10^–3^
*Oryza sativa*	grain	non-shattering	5700−3500 BC	26 269	86.7%	0.04%	3.6×10^–3^
*Lens culinaris*	grain	seed length	9450−6550 BC	1032	40.9%	0.02%	1.0×10^–3^
*Cucurbita pepo* (Mesoamerica)	ruderal	seed length	8500−6500 BC	15	16.0%	0.8%	0.9×10^–3^
*Cucumis melo* (long episode)	ruderal	seed width	3300−1900 BC	462	24.0%	0.02%	1.1×10^–3^
*Cucumis melo* (short episode)	ruderal	seed width	2500−2000 BC	308	23.0%	0.05%	2.6×10^–3^
*Capsicum baccatum* (long episode)	ruderal	seed area	4074 BC−1491 AD	70	77.7%	0.01%	0.8×10^–3^
*Capsicum baccatum* (long episode)	ruderal	seed area	300−1491 AD	48	22.2%	0.02%	3×10^–3^
*Secale cereale* (long episode)	segetal	grain breadth	1255−340 BC	133	25.9%	0.03%	2.2×10^–3^
*Secale cereale* (short episode)	segetal	grain breadth	1255−450 BC	30	41.2%	0.05%	6.5×10^–3^
*Paspalum scrobiculatum*	segetal	grain breadth	500−115 BC	123	54.0%	0.14%	5×10^–3^
*Prunus persica*	tree fruit	stone length	3500−1400 BC	143	35.2%	0.023%	4×10^–3^
*Olea europea*	tree fruit	stone length	4400−150 BC	824	59.6%	0.01%	0.5×10^–3^
*Phoenix dactylifera*	tree fruit	seed length	3000 BC—650 AD	1370	72.3%	0.02%	7.4×10^–3^

In terms of understanding the driver for this process, we must recognize that rice was challenged by the drier conditions of the Deccan region of peninsular India. Wild *Paspalum* is often a weed of rainfed rice, but may also occur in wet, deepwater rice. Rainfed rice was among the earliest rice cultivation systems in south India in areas with *ca* 900 mm annual rainfall [94]. Even if the minimum rainfall necessary to grow rice is considered to be 800 mm [104], such moisture levels would have supported very modest yields. What is more, the drier Deccan region is prone to drought years. Some sources suggest drought as often as 3 years in 10 may occur in south India [105]. In such circumstances reduced or failed rice productivity would have encouraged consumption of weedy *Paspalum* grain from rice fields, until some farmers chose to re-sow *Paspalum* as a more reliable crop in its own right.

## 6. Comparing durations and rates of domestication

Previous work has argued that grain domestication took place unintentionally through unconscious selection [3,4,40], whereas fruit trees more likely involved some element of intentional, conscious selection for improved size and taste [33]. Here, we compare the rates and lengths of episodes for these domestication pathways with those for ruderals and segetals (table 2).

The review of the archaeobotanical data for several ruderal and segetal domesticates suggest differences in process and rate. In the ruderal pathway, early use of wild fruits would have facilitated spontaneous seeding around areas of human activity. Where human occupations were continuous (sedentary) or seasonal but recurrent during the fruiting season, harvesting would have been facilitated by incidental stands adjacent to human occupation. The evolution of domestication traits via unconscious selection was relatively slow. However, the habitats in which they were growing were adjacent to human encampments and would have facilitated observation of phenotypic variability (such as size, colour, taste, and so on) which could have been further selected upon consciously when people chose to cultivate (to intentionally sow) seeds from these fruits. The generally slower Haldane rates, often comparable to primary grain pathway domestication rates estimated in previous work (table 2), suggest the ruderal pathway tended to be slow and driven by unconscious selection.

By contrast, the Haldane rates estimated for segetal pathways are several times faster than for cereals under the grain crop pathway. The increased rate of domestication and shorter domestication episodes suggest a greater role for conscious selection. Segetal crops may have become more significant in environments that were marginal for the main crops, and experienced farmers chose to switch to the new crops. Farmers would have already cultivated cereals and thus be able to adopt comparable practises for the cultivation of segetal weeds, thereby generating new cereal crops. Selection for larger grains and non-shattering might therefore have included some conscious drivers. The higher Haldane rates for segetal cereals are comparable to those estimated for several tree fruits, which were also intentionally selected for larger and sweeter fruits [33].

## 7. Conclusion

We have presented conceptual frameworks and archaeobotanical data to clarify two plant domestication pathways, ruderal and segetal, previously proposed in brief but not elaborated in detail. Ruderal crops were probably domesticated slowly, primarily through unconscious selection; whereas segetal crops were domesticated faster, primarily through conscious selection by established farming cultures. The rates and types of selection in the ruderal and segetal pathways are comparable to those in the cereal and fruit tree pathways, respectively.

We end by noting that agricultural diversity formed not just from early centres of domestication as usually defined (e.g. [4,106]), but increased in later periods as farmers experimented with potential new crops from anthropogenic habitats. Ruderal contexts provide potential cultivars not only from around hunter-gatherer camps, but even more so from around the more settled and anthropogenically transformed habitats of sedentary farming societies. These disturbed, anthropic environments provide diverse habitats for ruderal species to flourish, from which species were subsequently selected for more intensive management, potentially leading to cultivation. Segetal weeds are often portrayed negatively in terms of competing with crops and reducing crop yields, but they also offer a reservoir of biodiversity from which new crops can be selected. European rye and Indian kodo millet were both domesticated during the respective Iron Ages, and thus jumps in agrobiodiversity took place in later prehistory.

## Data Availability

All data are compiled in the electronic supplementary material [107].
